# Chromosomal positioning and epigenetic architecture influence DNA methylation patterns triggered by galactic cosmic radiation

**DOI:** 10.1038/s41598-024-51756-7

**Published:** 2024-01-15

**Authors:** Adrian Perdyan, Marcin Jąkalski, Monika Horbacz, Afshin Beheshti, Jakub Mieczkowski

**Affiliations:** 1 International Research Agenda 3P - Medicine Laboratory, Medical University of Gdansk, Marii Sklodowskiej Curie 3a, 80-210 Gdansk, Poland; 2 Department of Biology, Stanford University, Stanford, CA USA; 3 Space Biosciences Division, NASA Ames Research Center, Blue Marble Space Institute of Science, Moffett Field, CA 94035 USA; 4 Stanley Center for Psychiatric Research, Broad Institute of MIT and Harvard, Cambridge, MA USA

**Keywords:** Computational biology and bioinformatics, Molecular biology, Space physics

## Abstract

Despite surging interest in space travel in recent decades, the impacts of prolonged, elevated exposure to galactic cosmic radiation (GCR) on human health remain poorly understood. This form of ionizing radiation causes significant changes to biological systems including damage to DNA structure by altering epigenetic phenotype with emphasis on DNA methylation. Building on previous work by Kennedy et al. (Sci Rep 8(1): 6709. 10.1038/S41598-018-24755-8), we evaluated spatial DNA methylation patterns triggered by high-LET (^56^Fe, ^28^Si) and low-LET (X-ray) radiation and the influence of chromosome positioning and epigenetic architecture in distinct radial layers of cell nucleus. Next, we validated our results using gene expression data of mice irradiated with simulated GCR and JAXA astronauts. We showed that primarily ^56^Fe induces a persistent DNA methylation increase whereas ^28^Si and X-ray induce a decrease DNA methylation which is not persistent with time. Moreover, we highlighted the role of nuclear chromatin architecture in cell response to external radiation. In summary, our study provides novel insights towards epigenetic and transcriptomic response as well as chromatin multidimensional structure influence on galactic cosmic radiation damage.

## Introduction

With a growing interest in space travel^1^, it is pivotal to better understand how galactic cosmic radiation (GCR) impacts human health and how its detrimental effect can be minimized^2^. Ionizing radiation damages DNA structure by inducing double-stranded breaks, single-stranded breaks, base damage and changing epigenetic phenotype with emphasis on DNA methylation patterns^3,4^. During long exposure, these alterations are likely to increase the risk of cancer and degenerative diseases occurrence^5^.

Besides hydrogen and helium, GCR consists of heavier atomic nuclei with high linear energy transfer (high-LET) values which are of great danger for human health. Recently, Kennedy et al.^6^ investigated an effect of high-LET ^56^Fe, ^28^Si ions on DNA methylation patterns in comparison to low-LET X-ray appearing in terrestrial radiation. The researchers found that the methylation status of ^56^Fe irradiated CpG sites can distinguish normal tissue from lung adenocarcinomas and squamous cell lung carcinomas. Additionally, the researchers showed acute radiation-induced methylation changes which persisted over time with heritable imprint across epigenome. These findings where further investigated by Nwanaji-Enwerem et al.^7^ showing that ^56^Fe exposure was associated with accelerations in epiTOC2 epigenetic clock suggesting that DNA methylation may be a sensitive biomarker of high-LET ^56^Fe ion exposure.

Chromatin packages the eukaryotic genome through DNA folding ranging from single nucleosome to entire chromosome^8^. Negatively charged DNA is wrapped around nucleosomes consisting of two copies of four positively charged histone proteins^9^. In addition to the core chromatin components, histone-modifying enzymes may alter chromatin compaction by changing its accessibility. The accessibility level is connected with chromatin modifications. In particular, H3 lysine 4 tri-methylation (H3K4me3) and histone H3 lysine 27 tri-methylation (H3K27me3) were used to profile ‘active’ and ‘repressed’ promoters respectively. Furthermore, histone H3 lysine 9 tri-methylation (H3K9me3) was used to profile heterochromatin loci and histone H3 lysine 27 acetylation (H3K27ac) to profile enhancers^10^.

There were a few reports suggesting that high-LET^4^ and ultraviolet^11^ induced DNA damage may be affected by chromatin structure. However, until now there are no studies investigating a DNA methylation pattern induced by low-/high-LET and spatial chromosomal conformation capture to explore the effects of chromatin physical properties on irradiation outcome. Importantly, with the recent development of high-throughput capture chromatin confirmation methods (Hi-C)^12^ and advanced algorithms to simulate the three-dimensional structure of nuclei such as Chrom3D, it is now possible to address the irradiation effects associated with chromatin conformation^13^. Recently, to access the multi-dimensional chromatin interactions researches were using Hi-C data for compartmentalization dividing chromatin into compartments associated with gene expression regulation^14,15^.

For the purpose of our work, we used Hi-C data to create detailed nuclear division introducing five distinct radial nuclear layers. Additionally, we characterized each layer separately using various of epigenetic and transcriptomic features. Specifically, we explored an effect of high-LET and low-LET ions on methylation DNA patterns in human lung epithelial cells using a previously published dataset of Kennedy et al.^6^. Further, we tried to understand how the radiation penetrates cell nucleus and influences methylation levels in distinct layers of nucleus, as well as how spatial chromosomal conformation and epigenetic architecture influence DNA alterations triggered by external radiation. To achieve this, we used existing publicly available *in-vitro* and *in-vivo* data and performed the following analyses: (1) Hi-C analysis to check how three-dimensional structure of cell nucleus impacts response to irradiation. Additionally, we divided cell nucleus into five laterally distributed layers to identify potential irradiation effects associated with peripheral and internal positioning of chromatin and other epigenetic features; (2) chromatin immunoprecipitation sequencing (ChIP-seq) data to assess the impact of distinct histone modifications on DNA methylation changes and (3) microarrays, and RNA-seq data to assess the impact of gene expression on irradiation response.

This research addresses the pressing need to understand the impact of galactic cosmic radiation (GCR) on human health, driven by the increasing interest in space travel. Our study builds on existing knowledge, investigating the influence of DNA structure on radiation outcomes. We used the concept of radial nuclear layers to explore how chromatin properties, including histone modifications and spatial conformation, affect DNA methylation alterations induced by low- and high-LET ions. Leveraging recent technological advances, such as high-throughput capture chromatin confirmation, we created a nuanced nuclear division with five nonoverlapping nuclear layers to better understand the spatial distribution of chromatin and its features, as well as its impact on radiation response. Our multiomics approach integrates Hi-C, ChIP-seq, and microarray/RNA-seq data, providing insights into the complex interplay between chromatin architecture, epigenetic modifications, gene expression and the cellular response to GCR. This research aims to contribute valuable information for developing strategies to minimize the adverse effects of cosmic radiation during space travel.

## Materials and methods

### Data description

**Figure 1:**
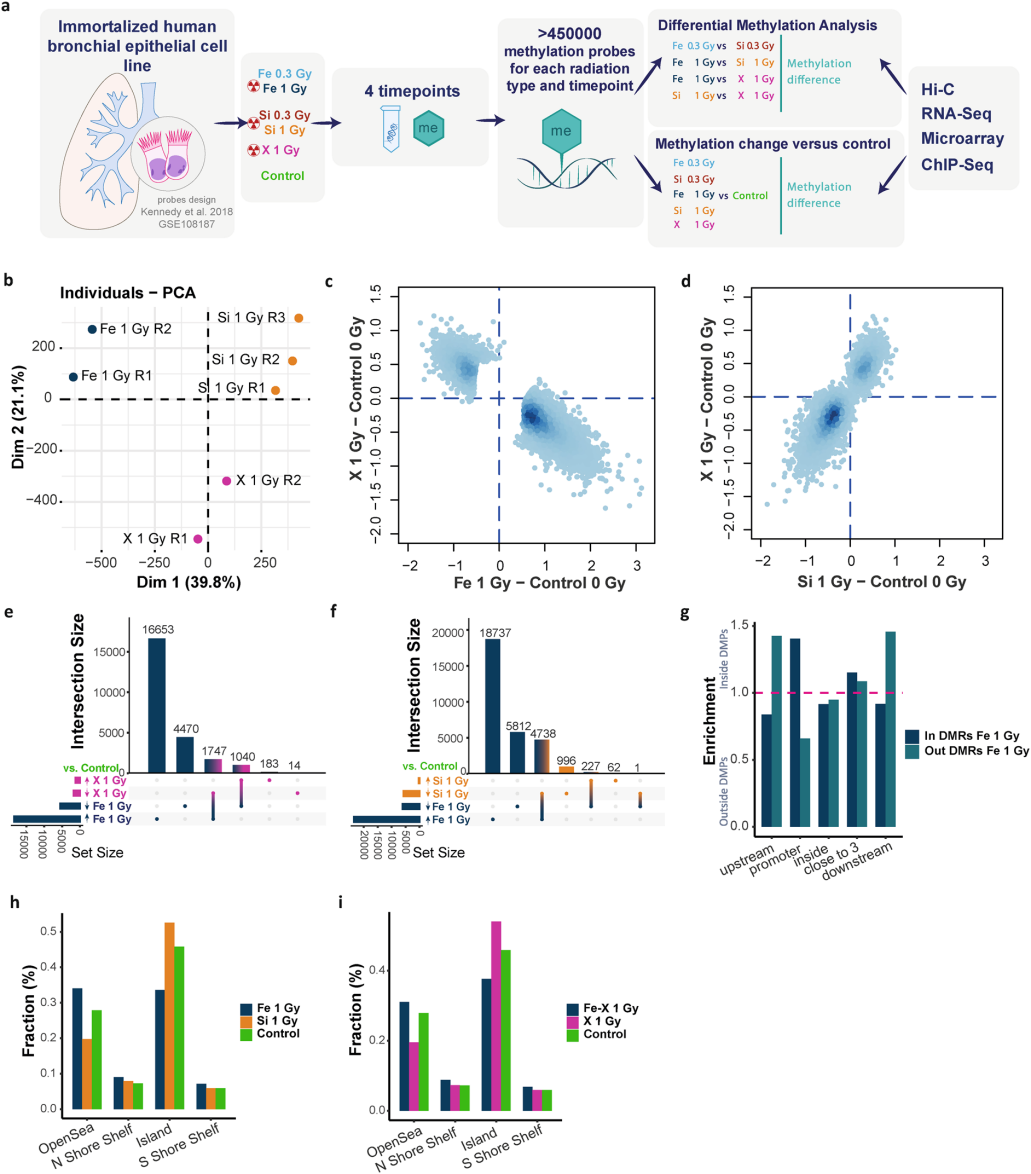
DNA methylation patterns and differential methylation analysis for 48-h post-exposure samples. (**A**) The schematic representation of the study methodology. (**B**) Principal component analysis (PCA) for 48-h post-radiation replicates. (**C**) DNA methylation change versus control among differentially methylated probes (DMPs) with applied filters (q-value (q < 0.05) and the absolute average difference (avDiff) between compared samples (avDiff ≥ 0.58)). (D) DNA methylation change versus control among common DMPs for Si 1 Gy and X ray particles from FeSi 1 Gy and FeX 1 Gy comparisons. (**E**,**F**) The overlay of DMPs which methylation level changed ( ≥ 0.58) versus control between particles. X axis—grouping of individual and overlayed DMPs; Y axis—number of DMPs in each group. (G) Genic location of DMPs located inside or outside differentially methylated regions (DMRs) (**H**,**I**) Relation of DMPs and primary probes to CpG islands. Shores are up to 2 kb from the CpG island; Shelves are from 2 to 4 kb from the CpG island; Opensea refers to isolated regions that do not have a specific designation. For the purpose of the analysis, we combined shores and shelves regions.

The study was conducted based on the publicly available NCBI GEO Methylation450K BeadChip human bronchial epithelial cell dataset published by Kennedy et al. in 2018 (Series GSE108187 on “https://www.ncbi.nlm.nih.gov/geo”; The National Aeronautics and Space Administration (NASA) GeneLab repository within the Open Science Data Repository (OSDR)https://genelab-data.ndc.nasa.gov/genelab/accession/GLDS-317/) (Fig. 1A)^6^. The dataset was comprised of 102 samples of immortalized human bronchial epithelial cell line (HBEC3-KT) established by introducing mouse Cdk4 and hTERT into normal human bronchial epithelial cells (HBECs)^16^. Cells were grown in a 5% CO_2_ environment at 37 °C and passaged (1:4) twice per week for three months. Three biological replicate cultures were irradiated independently with 0, 0.1, 0.3 or 1.0 Gy (Gy) ^56^Fe ions (LET = 170 keV/µm, Beam energy = 600 MeV/u) or with 0.0, 0.3, 1.0 Gy ^28^Si ions (LET = 70 keV/µm, Beam energy = 300 MeV/u) or 0.0, 1.0 Gy X-ray (LET = 2 keV/µm, Beam energy = 320 kV). Detailed information about the protocol can be found in the original publication^6^.

### Differential methylation analysis

To investigate the overall influence of each radiation type on methylation DNA patterns we established three pair comparisons between 1) ^56^Fe 0.3 Gy versus ^28^Si 0.3 Gy; 2) ^56^Fe 1 Gy versus ^28^Si 1 Gy; 3) ^56^Fe 1 Gy versus X 1 Gy. Before the analysis, we transformed provided in the original publication Beta Values (ratio of the methylated probe intensity and the overall intensity) into M Values (log2 ratio of the intensities of methylated probe versus unmethylated probe)^17^. Afterwards, we performed a differential methylation analysis of 485,061 probes for established comparisons using dmpFinder from Minfi Bioconductor package^18^. Differentially methylated probes (DMPs) were filtered based on the q-value (q < 0.05) and the absolute average difference in M Value between compared samples (absolute average difference ≥ 0.58). Additionally, we applied a threshold of M Value change versus control (absolute average difference  ≥ 0.58)^17^. Filtered DMPs were grouped into differentially methylated regions (DMRs) using dmrcate (lambda = 1000, C = 2) from DMRcate Bioconductor package^19^. The average methylation level for each DMR was calculated by overlapping filtered and ion specific DMPs. Due to the fact that culturing, irradiation, collection time (48-h) and DNA methylation levels were the same for all non-irradiated replicates, control DNA methylation levels were defined as average M Value of combined ^56^Fe, ^28^Si, X-ray non-irradiated samples (Fig. S1A). All analysis were performed in R Studio^20^. Mann–Kendall test was used for trend analysis with at least 4 data points. Chi-squared test was used to check differences between examined groups.

### Chromatin conformation capture (Hi-C)

To investigate the influence of chromosomal conformation on the effect of radiation we used the Hi-C data of IMR-90 human lung fibroblast cell line published by Rao et al. in 2014 (Series 4DNFIHM89EGL; 4DNFIMNT2VYL on “https://data.4dnucleome.org/”)^12^. Based on the provided Hi-C score, the nucleus was divided into five distinct layers named from the most outer “L1” to the deepest “L5”. The nucleus layers were created by overlapping all probes from the primary experiment^6^ with Hi-C data^12^. Layers boundaries were assigned quantile 0%, 20%, 40%, 60%, 80% and 100% values of Hi-C score. Hence, each layer contained the same amount of DNA. Accordingly, to investigate spatial gene expression patterns in Japan Aerospace Exploration Agency (JAXA) astronauts’ blood tissue^21^, we used conserved datasets of K562 and HAP-1 human lymphoblasts isolated from chronic myeloid leukemia patient and induced pluripotent stem cells (Series 4DNFI5WH9HQX; 4DNFIHM89EGL; 4DNFIOZT9QGF on “https://data.4dnucleome.org/”, respectively). Genes which were differentially expressed (see methods for details) in all datasets among distinct nucleus layers were further analyzed^22–25^.

### Histone modifications

To investigate active (H3K27ac, H3K4me3) and repressive (H3K9me3, H3K27me3) regions of chromatin we assigned different histone modifications to identified DMPs. We used the control datasets of primary human bronchial epithelial cells published by Stolzenburg et al. in 2017^26^ and Mazumdar et al.^27^ in 2020 (Series GSE94726, GSE132807 on “https://www.ncbi.nlm.nih.gov/geo”, respectively). Each dataset was filtered to include peaks from 300 to 2000 base pairs long. Additionally, GSE132807 was filtered based on the alignment score from Bowtie2 (score >  = 10) to asses high signal peaks^28^. For GSE94726 Series, alignment score of > 0 from Bowtie 1 was used^29^.

### Gene expression validation

To validate the in vitro model, we used processed RNA-sequencing (Series OD-203, OSD-294, OSD-530) and microarray (Series OSD-80, OSD-109, OSD-117, OSD-159) gene expression data of whole-body irradiated (no direct information provided for OSD-294 and OSD-80) mice and JAXA astronauts provided with courtesy by the NASA GeneLab Data Repository (https://genelab-data.ndc.nasa.gov/genelab/projects/). Specifically, a variety of mice tissues irradiated with distinct particles were examined including liver (^56^Fe 0.2 Gy)^30^, retina (^57^Co 0.04 Gy) ^31^, heart (^56^Fe 0.15 Gy; ^1^H 0.9 Gy)^32^, mammary gland (Si 0.3 Gy, sparsely ionizing gamma rays 1 Gy)^33^ and blood (^137^Cs 2.7 Gy)^34^. Additionally, we examined blood samples of six JAXA astronauts^21^. Due to the astronauts’ data provided only by mean expression values of all astronauts, there was only one replicate per distinct timepoint. Hence, to perform differential expression analysis, we established five distinct timepoints using two following timepoints as replicates [1) Control—L-112/56(± 14d) day – pre-flight; 2) L + 5(± 1d) day, and L + 30(± 7d) day—in-flight; 3) L + 60/120(± 14d) day or R-8(-14d/ + 0d) day—in-flight; 4) R + 3(± 1d) day, and R + 30(± 7d) day—post-flight; 5) R + 60/120(± 14d) day—post-flight; L-launch; R-rescue]. To establish research models for differential expression analysis, we built the EdgeR object using limma package^35^. We normalized RNA-sequencing data using weighted trimmed average of the log expression ratios, and performed differential analysis fitting a negative binomial generalized log-linear model. For microarrays, we used already normalized data with median intensity threshold of >  = 3^36^ and applied single-channel experimental design for several groups with Bonferroni adjustment. For further gene expression, we included genes with false discovery rate (FDR) < 0.05 in the first time point, and used log fold-change (logFC) score in the following timepoints to assess the gene expression chronic change. Up- or down-regulated genes were defined as increase or decrease of logFC in the first timepoint versus control, respectively. For trend analysis, we performed an anova test on linear regression gradient.

## Results

### Ion-specific effects on DNA methylation profile 48 h after irradiation

To analyze DNA methylation patterns caused by high-LET (^56^Fe, ^28^Si) and low-LET (X-ray) participles, we used publicly available data set^6^. The schematic representation of performed analysis is shown in Fig. 1A. By applying differential methylation analysis on 48-h post irradiation samples, we identified 26,751, 31,818 and 24,807 differentially methylated probes (DMPs) for ^56^Fe 0.3 Gy versus ^28^Si 0.3 Gy; ^56^Fe 1 Gy versus ^28^Si 1 Gy; ^56^Fe 1 Gy versus X 1 Gy, pair comparisons, respectively (see methods for details). We did not identify any significant DMPs for ^28^Si 1 Gy versus X 1 Gy pair comparison (adjusted p-value > 0.05), suggesting their similar effect (Fig. 1B). Similarly, to Kennedy et al.^6^, we observed that regardless of the dose, ^56^Fe predominantly increases DNA methylation whereas X ray decrease DNA methylation levels when compared to control. However, contrary to primary article where no effect of ^28^Si was observed, we discovered its activity in decreasing DNA methylation levels (Fig. 1C,D, S1B,C). It could happen due to different hypothesis testing in the original article, where authors were focused on DNA methylation changes caused by an increase in the dose of the same radiation. The opposite methylation effect between ^56^Fe and ^28^Si or X-rays was observed nearly among all identified DMPs for all comparisons (Fig. 1E,F, S1D).

### The distribution of differentially methylated probes indicates a stochastic process

To further identify the distinct effect of each particle on DNA methylation patterns, in addition, to direction of methylation change (Fig. 1E,F, S1D) we divided DMPs into distinct subgroups, based on post-exposure and control M value (hypomethylation [M Value < 0] or hypermethylation [M Value > 0]; see methods for details). Specifically, we showed that ^56^Fe was able to induce, both hypermethylation of primarily hypomethylated sites and hypomethylation of primarily hypermethylated sites whereas ^28^Si induced only the latter (Fig. S1E,F). Accordingly, X-ray induces hypomethylation of previously hypermethylated sites, however, it does have a minor effect on hypomethylated sites inducing its hypermethylation. In summary, when comparing post-radiation to control DNA methylation levels, ^56^Fe affects hypomethylated and hypermethylated sites whereas ^28^Si is more likely to affect hypomethylated sites at control.

Further, to check whether DNA methylation changes occur in a close relationship to each other and around the radiation beam, we built differentially methylation regions (DMRs) from previously identified paired DMPs (Fig. S1G). In total, we identified 3805, 4863 and 3631 DMRs for ^56^Fe 0.3 Gy versus ^28^Si 0.3 Gy; ^56^Fe 1 Gy versus ^28^Si 1 Gy; ^56^Fe 1 Gy versus X 1 Gy, pair comparisons, respectively. Interestingly, only 41%, 47% and 43% of DMPs built DMRs, respectively, leaving more than 50% of identified DMPs outside of DMRs (Fig. S1H). As such, we applied a larger base pair limit for DMRs building, however, did not observe a relevant change in amount of DMRs (Fig. S1I). Finally, by permutating methylation probe names from primary dataset, we simulated differential DNA methylation analysis in one thousand unique datasets. In all replicates, we observed lower frequencies of DMPs building DMRs when compared to primarily established comparisons (*p* < 0.001). This observation suggested that the distribution of radiation induced DNA methylation changes seems to be spread across the whole genome rather than being grouped in DMRs.

### Assignment of differentially methylated probes (DMPs) to genomic location

Following previous results, to investigate specific genomic locations of DMPs, we looked at the relation to CpG islands localized in the close relationship of gene promoters^18^. We found that, regardless of radiation type and dose, grouped DMPs were more likely located inside promoter regions (Fig. 1G; *p* < 0.001 for all particles and doses; data not shown). It suggests that proximal regulatory regions of human genome are more prone to accumulate radiation induced DNA methylation changes. Additionally, the enrichment in upstream and downstream locations for DMPs outside of DMRs showed that when looking only at DMRs, we would lose an important regulatory information. Next, we checked if this observation could be due to a higher accumulation of CpG rich regions in the corresponding promoters or higher accumulation of primary probes in these locations. However, when comparing fractions (ratio of a component to the total amount) of post-exposure samples to control, we observed distinct patterns, especially in terms of CpG islands and regulatory opensea regions (Fig. 1H,I; *p* < 0.001 for all comparisons)^37^. Finally, we observed, that regardless of radiation dose, DMPs inside DMRs were more likely to be located inside CpG islands which are known to be positioned in close relationship of promoters (Fig. S1J–L)^38,39^. Additionally, when taking into consideration the direction of methylation change, CpG islands were more prevalent among sites which methylation increased after ^56^Fe or decreased after ^28^Si and X exposure (Fig. S1J–L). In summary, these observations indicate that ^56^Fe primary increases DNA methylation, ^28^Si decreases DNA methylation whereas X-rays induce minor effect in both directions. Moreover, radiation induced DMPs are primary located around active promoter regions and opensea regulatory regions.

### Radial chromosomal distribution in nucleus affects changes in DNA methylation profiles after irradiation

**Figure 2:**
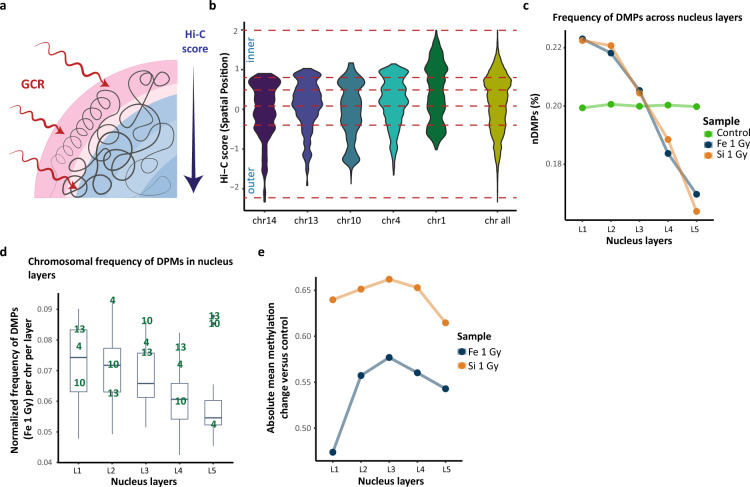
Radial nucleus architecture and DNA methylation patterns. (**A**) The schematic representation of nucleus division based on the high-throughput chromosome conformation capture (Hi-C) technique score. Layers boundaries were assigned quantile 0%, 20%, 40%, 60%, 80% and 100% values of Hi-C score of all raw probes used in the primary experiment. Hence, each layer contains the same amount of DNA, but can vary in width. (**B**) Chromosome positioning of non-irradiated primary probes among five nucleus layers (dashed red lines). Chromosomes 13, 10 and 4 have the highest 48-h DMPs frequency after Fe 1 Gy exposure. Y axis represents a Hi-C score for individual DMP. (**C**) The frequency of DMPs and non-irradiated probes among nucleus layers. (**D**) Chromosomal frequencies of DMPs in each nucleus layer (data shown for Fe 1 Gy exposure). Top three chromosomes with the highest overall DMPs frequency are bolded. Boxplots represent data for whole genome. (**E**) Absolute mean DNA methylation change in DMPs versus control among nucleus layers.

Further, we investigated whether DNA methylation changes are conditioned by the epigenetic state and chromatin physical properties. We studied the induced DNA methylation changes in the context of 3D chromatin structure and histone modification profiles in untreated cells. We overlapped Hi-C compartment coordinates of IMR-90 human lung fibroblast cell line provided by Rao et al.^12^ with baseline probes analyzed previously^6^. We identified 430,748 probes to which we assigned a compartment Hi-C score which determines the positioning of certain probes in cell nucleus. The greater the score is, the deeper into nucleus a certain probe should be located (Fig. 2A)^40^. As such, we drew an approximate radial positioning of each chromosome in the nucleus and later investigated its impact on the frequency of DNA methylation sites (Fig. S2A). First, we normalized the number of DMPs on each chromosome with regard to the total number of probes on each chromosome used in the experiment. This way corrected for bias associated with the design of the microarray. The highest DMPs prevalence was observed on chromosomes 4, 13, 10 for ^56^Fe (Fig. 2B; Fig. S2B–D); 4, 2, 18, 14 for ^28^Si (Fig. S2E,F) and 13, 18, 15 for X (Fig. S2G).

To investigate whether the observed DMP frequencies depend on the 3D chromatin structure we divided the nucleus into five distinct layers as shown on Fig. 2B and S2A (see methods for details). Nucleus layers were created based on the even distribution of all DNA methylation probes in order to include the same amount of DNA within each layer. On the graph, we showed the frequency percentage of all DMPs in each layer separately to visualize where DMPs are the most prevalent. Accordingly, for each radiation type we observed the highest prevalence (% of DMPs in a layer/all DMPs) of DMPs among outer “L1” and “L2” layers with a significant decrease towards the depth of nucleus (*p* = 0.027; Fig. 2C, S2H). As such, we looked at the chromosomal frequencies in each layer separately. We discovered that chromosomes with the highest DMPs frequency are cumulating DNA methylation sites at the “L2”–“L3” junction and among the following layers (Fig. 2D; S2I–M). Thus, in the outer “L1” layer the effect of radiation seemed to be limited and resulting in a more spread distribution of DMPs. The corresponding chromosomal frequencies patterns were observed in deeper layers of the nucleus starting at “L2”–“L3” junction.

Accordingly, we looked at the absolute methylation change across distinct layers. We discovered the highest DNA methylation change being present around the middle “L3” layer (Fig. 2E; S2N). Interestingly, the methylation change induced by ^56^Fe persisted until the “L5” having the lowest value in “L1” whereas the effect of ^28^Si and—ray seemed to be limited having the lowest change in “L5”. In summary, these observations could suggest that outer layers of nucleus absorb the highest radiation dose, thus having more differently methylated sites, however, they are randomly distributed across all chromosomes without a particular pattern. Interestingly, despite high frequency of DMPs in outer layers, the average methylation change peaks in the middle “L3” layer which may suggest other factors limiting the effect of GCR radiation in outer layers of nucleus.

### Irradiation induced DNA methylation patterns are associated with histone modification

**Figure 3:**
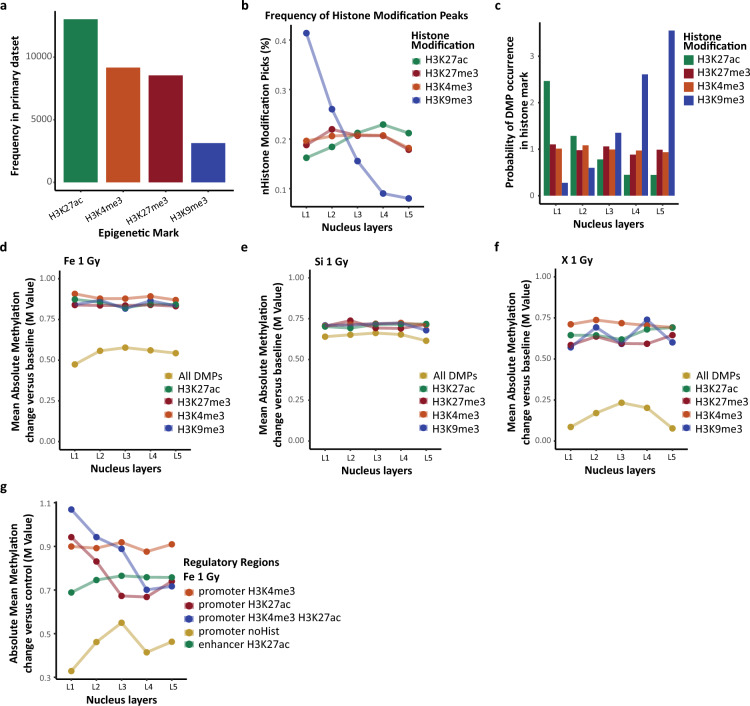
Radial nucleus architecture of histone modifications. (**A**) The frequency of distinct histone modifications among non-irradiated primary probes. (**B**) The distribution of distinct histone modifications among non-irradiated primary probes within nucleus radial layers. (**C**) The probability of DMP overlapping distinct histone modification peak in each nucleus layer. (**D**–**F**) Absolute mean DNA methylation change versus control among histone modification peaks overlapped with DMPs within nucleus layers. (**G**) Absolute mean DNA methylation change versus control among histone modification associated regulatory regions overlapped with DMPs within nucleus layers.

Next, we explored how chromatin modifications predispose cells to particular irradiation response, and we looked at the distribution of histone modifications. Specifically, we examined four histone modifications (H3K4me3, H3K27ac, H3K27me3, H3K9me3) which are known to have a major role in chromatin organization. We used enrichment profiles published by Stolzenburg et al.^26^ and Mazumdar et al. ^27^ (see methods for details). When overlapped filtered histone modifications peaks with primary probes, euchromatin-associated histone modifications were more frequent than heterochromatin-associated ones, however, it may be due to distinct primary datasets used in this study (Fig. 3A). Comparing between nucleus layers, we observed the enrichment of H3K9me3 among external layers with a decreasing towards inner nucleus layers. That distribution was contrary to other histone modifications which level did not depend on the layer (H3K4me3, H3K27me3) or increased (H3K27ac; Fig. 3B). Further, we examined the probability of DMP occurrence ([DMP in regulatory regions/all DMP]/[regulatory regions in layers/all regulatory regions]) in distinct histone marks among nucleus layers. Interestingly, regardless of radiation type and dose, we found a decreasing trend for H3K27ac (*p* < 0.05), an increasing one for H3K9me3 (*p* < 0.05) and no trend for H3K4me3 and H3K27me3 (*p* > 0.05; Fig. 3C; data shown for ^56^Fe 1 Gy).

Looking at the spatial baseline average methylation level inside histone modifications picks, heterochromatin-associated marks (H3K9me3, H3K27me3) had higher baseline methylation levels than euchromatin-associated marks (H3K27ac, H3K4me3; Fig. S4A). Specifically, as for H3K27ac and H3K4me3, the methylation level was rather stable (*p* = 0.22; *p* = 0.087, respectively) across distinct nucleus layers whereas among H3K9me3, H3K27me3 a methylation increase was observed towards the inner layers (*p* = 0.027). Next, we checked for absolute methylation changes (vs. control) induced by irradiation in epigenetic modifications. We found that, regardless of radiation type and dose, the average absolute methylation change in each mark was rather stable, without a particular trend observed between nucleus layers (*p* > 0.05; Fig. 3D–F; S3B–D). However, when compared the mean absolute methylation change to all DMPs, we discovered that the methylation change was higher in all histone marks, regardless of radiation type and dose (except Si 0.3 Gy; Fig. 3D–F; S3B–D).

Furthermore, to investigate the DNA methylation patterns in regulatory elements, we overlapped transcription starting sites (TSS) regions with H3K4me3 and H3K27ac histone marks using GENCODE annotation^41^. Additionally, we examined H3K27ac associated gene enhancers outside of TSS and gene bodies. In total, we included five distinct groups of regions in the analysis: (1) H3K4me3-associated promoters; (2) H3K27ac-associated promoters; (3) H3K4me3- and H3K27ac-associated promoters; (4) no histone associated promoters; (5) H3K27ac-associated enhancers.

As expected, when looking at the spatial frequency of these regions, we observed higher prevalence of promoters and enhancers in deeper layers of nucleus which was contrary to the no histone associated regions (Fig. S3E). Next, we checked for spatial frequencies of primary probes overlapping them with previously established regions. We observed a strong increasing trend towards inner parts of nucleus for all regulatory regions (*p* < 0.05; Fig. S3F). Moreover, the baseline methylation levels of histone associated regulatory regions overlapped with primary probes were much lower than those not associated with any histone mark (Fig. S3G). Next, we checked for absolute methylation change in DMPs which were overlapping these regions. Interestingly, the highest observed methylation change was observed in outer nucleus layers among H3K4me3 and/or H3K27ac associated regions (Fig. 3G; S3H–L). Specifically, for most prevalent ^56^Fe irradiated samples the highest methylation change was observed in “L1” and “L2” layers in both H3K4me3 and H3K27ac associated promoters (Fig. 3G). More importantly, when comparing an absolute mean methylation change between regulatory regions and previously established samples (Fig. 3D–F), we discovered that in general methylation change was higher in histone mark associated sites, especially among regulatory regions. In summary, these findings show that promoters and enhancers are prone to GCR induced DNA methylation changes. Moreover, H3K4me3 and H3K27ac histone marks are associated with the highest DNA methylation change, suggesting its vulnerability to irradiation. To summarize, genomic regions prevalent with distinct histone marks, especially regulatory regions are associated with higher methylation changes. Interestingly, the highest changes are observed in outer layers of nucleus. Hence, all these results may highlight the role of histone modifications in response to external radiation, for instance, by protecting inner euchromatin-associated regions. On the other hand, regulatory regions associated with histone marks are more prone to accumulate radiation associated damage with regard to DNA methylation.

### Chronic (> 48 h) DNA methylation patterns of various radiation types in in-vitro and in vivo models

**Figure 4:**
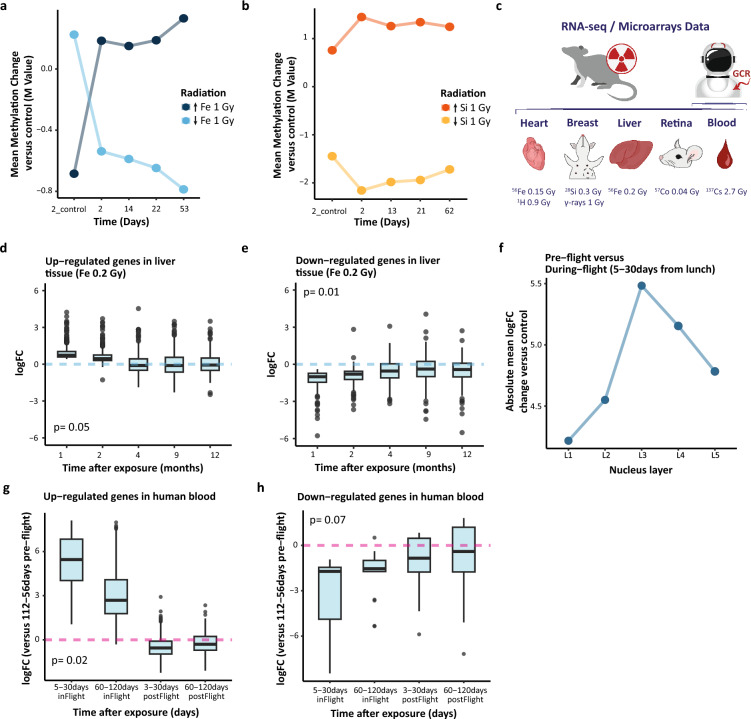
Persistence of DNA methylation and gene expression change over time. (**A**,**B**) Mean DNA methylation change versus control over time with inclusion of direction of methylation change. (**C**) The schematic representation of gene expression data used to check for corresponding chronic patterns. (**D**,**E**) The average change of gene expression in mice liver over time among up-regulated and down-regulated genes. (**F**) The absolute mean gene expression changes among nucleus layers for differentially expressed genes. (**G**,**H**) The average change of change expression in astronauts’ blood over time among up-regulated and down-regulated genes.

Finally, to further explore the DNA methylation changes induced by the selected irradiation, we looked at the methylation change over time. We discovered that regardless of the dose and the direction of methylation change ^56^Fe induces persistent DNA methylation changes (Fig. 4A; S4A). On the other side, ^28^Si and X induce DNA methylation changes which are not durable, hence the post-exposure methylation level steadily comes back towards the baseline level (Fig. 4B; S4A). To check if similar effect is observed on functional level, we used gene expression data of the NASA’s OSDR (see methods for details). We examined heart, breast, liver, retina and blood tissues using *in-vivo* and RNA-sequencing and microarrays data of whole-body irradiated mice and blood tissue RNA-sequencing of six JAXA astronauts (Fig. 4C). Specifically, we did not juxtapose DNA methylation results, as we looked only at the gene expression patterns of differentially expressed genes over time. Hence, we wanted to verify if the previously observed radiation effect is also observed on the transcriptomic level.

Accordingly, we observed corresponding long-term gene expression patterns in mice liver tissue, irradiated with ^56^Fe 0.2 Gy particles. For both the sets up-regulated and down-regulated genes the change of average gene expression level was the highest a month after irradiation and later stabilized, and persisted up to 12 months (*p* = 0.05, *p* = 0.01, respectively; Fig. 4D,E). On the other side, in microarrays data, we observed corresponding short-term gene expression patterns in mice heart tissue, irradiated with ^56^Fe 0.15 Gy particles (*p* = 0.33 and *p* = 0.17, respectively; Fig. S4B-C). We also examined, long-term gene expression patterns in mice breast tissue, irradiated with ^28^Si 0.3 Gy particles and observed corresponding, however not significant, pattern to our in vitro analysis. Hence, fluctuating towards baseline level in the last point of observation (Fig. S4D,E). Finally, to check if observed expression patterns are specific for each radiation type, we investigated the chronic effect of protons, gamma rays, ^137^Cs and ^57^Co, exposing distinct gene expression patterns over time (Fig. S4F–L; see method for details).

Lastly, we checked spatial and long-term gene expression patterns in blood tissue of JAXA astronauts (see methods for details). Specifically, among nucleus layers we checked if we observe a corresponding pattern in gene expression to DNA methylation change right after astronauts were sent to space and exposed to GCR and if it persists during and after their mission in space. Importantly, we observed that the change of gene expression peaks in the middle “L3” nucleus layer for differentially expressed and all identified genes (Fig. 4F; S4M, respectively), which stands in line with previous DNA methylation observations (Fig. 2E). Moreover, we discovered corresponding long-term persistence of gene expression change for up-regulated (*p* = 0.02) and down-regulated genes (*p* = 0.07; Fig. 4G,H). Specifically, we observed increase or decrease of gene expression right after launch and during the mission, as well as effect persistence up to 3–4 months (the last timepoint) after earth landing.

In summary, despite variety of timepoints used when compared to DNA methylation study, we saw corresponding trends in gene expression patterns in several mice tissues and astronauts’ blood tissue. Hence, we observed a gene expression change right after irradiation exposure, however, the epigenome imprint was persistent for ^56^Fe or space exposure when compared with ^28^Si. As such, we confirmed the previous DNA methylation findings on functional level. Lastly, we checked for DNA methylation change persistence over time in histone modification peaks, promoters and enhancers regions overlapped with DMPs and observed corresponding results to previously established patterns (data not shown).

## Discussion

This is the first study exposing influence of chromatin physical properties on DNA methylation patterns triggered by high-LET and low-LET radiation. Our work shows that each of investigated radiation types induce distinct DNA methylation patterns. Additionally, we exposed the major role of spatial chromosomal positioning and distribution of histone modifications which have undeniable influence on the effect of cosmic radiation on human cells. Finally, we validated our in vitro observations regarding chronic effect of high-LET particles, with in vivo models and astronauts’ data. As such, we supported our primary analysis with gene expression data of radiation exposed mice tissues including liver, heart, mammary gland, blood and retina as well as blood tissue from six JAXA astronauts’.

Our findings partially support those reported in the original publication by Kennedy et al.^6^. Similarly, we showed that each of primarily investigated radiation types induce a unique imprint on the epigenome. However, we observed that methylation changes which occur early, persist over time only when caused by ^56^Fe particle. Thus, after ^28^Si and X-rays’ exposure, we found a methylation peak at the beginning and a decrease towards baseline level over time. In line to previous observations a vast majority of identified methylation sites was located in and around CpG islands, and active genic regions (promoters, enhancers). The differences in results when compared to original publication could be of a various reason^6^. Specifically, we focused on rigorously chosen differentially methylated sites applying several thresholds of significance. To asses this, we established pairs to compare particles between each other. On the other side, in primary publication authors were focused on global DNA methylation status including all probes without DNA methylation change thresholds and later on assessing differences only by comparison of doses within the individual particle. To support our approach, we validated the perseverance of epigenome imprints associated with distinct radiation exposure. To do this, we looked at gene expression of various mice tissues irradiated with different radiation types as well as JAXA astronauts data from blood liquid biopsies^21,30–34^. Collectively, our analysis confirmed that each of investigated radiation type induces a unique chronic gene expression pattern. Importantly, we found corresponding changes between DNA methylation and gene expression levels in terms of GCR particles as well as astronauts’ exposure to space radiation. Thus, however to properly examine the persistence of these patterns, multiple timepoints with longer period of observation would be preferred. Additionally, all experiments should be performed in the same research model to minimize potential heterogeneity among even similar cell lines or more advanced models.

Interestingly, in recent years other researchers investigated both DNA methylome and transcriptome changes in several tissues exposed to spaceflight. In retinas of mice flown to space for 37-days period, Chen et al. found a large number of differentially methylated (DM) genes and fewer differentially expressed genes at the transcriptome level. Majority of DM sites were hypomethylated and located in genes and promoters. The functional analysis of these data revealed that spaceflight had a major effect on extracellular matrix/cell junction and cell proliferation/apoptosis signaling^42^. On the other side, Acharya et al. demonstrated increased DNA methylation levels in the hippocampus and ultimately impaired cognitive condition of irradiated mice which could be mitigated by 5-iodotubercidin administration^43^. Finally, in NASA Twins Study, despite observing minor genome-wide DNA methylation changes, the gene enrichment analysis showed that during flight genes involved in the response to platelet-derived growth factor and T cell differentiation, and activation pathways in both CD4 and CF8 cells were enriched. Specifically, for CD4 cells genes involved in platelet aggregation, regulation of ossification, and cellular response to UV-B were enriched, whereas for CD8 cells genes involved in somatostatin signaling pathway and positive regulation of superoxide anion generation were enriched^44^.

To further investigate the methylation data, our analyses focused on the impact of spatial chromosomal positioning on DNA methylation patterns or radiation properties and in vivo validation of induced epigenome imprints^45^. At first, based on spatial chromosome distribution, we tried to establish features associated with DMPs prevalence. At the beginning, we associated the higher frequency with outer positioning of chromosomal fragments in “L1” layer and its absence in “L5” layer. Hence, suggesting that when penetrating the nucleus, externally exposed DNA fragments are at the highest exposure to radiation beams which effect decreases with nucleus depth^4^. This observation however seemed promising, was not persistent for the whole genome, proposing the influence of other unknown factors. Recently, Garcia-Nieto et al.^11^, demonstrated that epigenome architecture in ultra violet (UV) irradiated non-malignant lung fibroblasts cells plays a role of a barrier to cancer development by regulation of carcinogen susceptibility. These findings highlighted the role of H3K9me3 modification in accumulating UV-induced DNA lesions in external heterochromatin regions, thus protecting inner localized euchromatin. As such, we looked at the spatial distribution of epigenetic histone modifications which may also play a role in the context of GCR^11,46^. Likewise, we observed the highest prevalence of DMPs in the periphery with the highest DNA methylation changes among active promoter and enhancers regions marked with H3K4me3 and H3K27ac histone modifications. However, when looking at all identified DMPs the highest methylation change was observed around the middle layer, right after the euchromatin-associated histone marks overcame the heterochromatin-associated H3K9me3 histone mark. Additionally, from that point, we started to observe a similar DMP chromosomal frequency pattern, suggesting the higher radiation vulnerability to occur differentially methylated sites as well as higher DNA methylation changes, especially among internally located promoters and enhancers sites. Specifically, this could be associated with higher chromatin accessibility.

As previously mentioned, at first, we hypothesized that nucleus periphery absorbs the highest radiation dose. However, despite the highest frequency of DMPs observed, the highest DNA methylation change and corresponding chromosomal frequencies were observed around the middle layer of the nucleus. It could potentially suggest the occurrence of Bragg peak which depicts the energy loss of ionizing radiation during its travel through matter and after which the after which particles immediately come to rest^47^. It might be true especially for ^28^Si and X-rays, since we observed a higher DNA methylation change drop in inner layers for these two types of radiation. However, promising in principle X-rays penetrate the whole nucleus and lose energy in a random manner along the photon path, and not having Bragg peak present due to presence of uncharged photons in X rays. Additionally, DNA double strand breaks (DSBs), labeled as gH2AX foci, are distributed “randomly,” without accumulation at the nuclear periphery, but more prevalent in nuclear compartments with low chromatin density^48,49^. Although, never investigated for ^56^Fe and ^28^Si particles, based on similar experiments performed using 1 Gy for ^11^B and 1.2 Gy for ^20^Ne (LET 150 keV/µm and 171 keV/µm, respectively; Energy beam 8 MeV/n and 47 MeV/n, respectively), we could suspect that all of investigated particles by Kennedy et al., should penetrate the whole nucleus width, therefore, it is unlikely to observe a Bragg peak in the middle and all other nuclear layers^50^. Hence, our results could be potentially associated with chromatin accessibility or specific properties of different radiation types used, rather than particular histone modifications occurrence.

Previous reports shown that the accumulation of double-strand breaks (DSBs) in peripheral heterochromatin does not accumulate more DSBs that euchromatin. Further, the exposure of cells to photonic radiation leads to a higher occurrence of DSBs in decondensed euchromatin. In contrast, exposure to high-LET results in more severe damage to dense heterochromatin. The distinct interaction of various types of radiation with different chromatin domains can be attributed to their diverse mechanisms of action. Photonic (gamma or X-ray) radiation primarily induces DNA damage indirectly through free radicals (ROS) generated by water radiolysis. Conversely, ions with high-LET directly damage DNA. Dense heterochromatin acts as a protective shield against ROS, yet it provides more DNA targets per volume for accelerated particles. Crucially, the extensive induction of DSBs by high-LET ions is not confined to peripheral heterochromatin but extends to the entire nucleus^50–52^. Hence, specific methylation changes may be generated as part of the systemic response of cells to irradiation. Indeed, post-irradiation epigenetic modifications have been shown not only to occur at sites of DSB damage, but also globally, throughout the cell nucleus^53^. Such a response can occur because chromatin represents a regulatory network and responses to various stresses as a system^54,55^. Hence, it seems more probable that these observations may be associated with chromatin accessibility and multidimensional architecture of these sequences rather than the epigenetic modification itself that makes H3K4me3 and H3K27ac-marked sequences more susceptible to radiation. Interestingly, Caron et al., found that most DMPs were located in chromosomes with low gene expression and located more peripherally compared to chromosomes with high gene expression. Thus, the intensity of DNA methylation changes correlates with both the low-expression (heterochromatic) state of chromatin and the peripheral location of chromatin. In our case, several of the few chromosomes with the highest abundance of DMPs are acrocentric chromosomes (12, 14, and 15), which also form a rim of heterochromatin around the whole width of the nucleus. It could indicate that heterochromatin architecture is a more important factor determining the intensity of DNA methylation changes after irradiation than nuclear location. Additionally, among heterochromatin layers, the highest DNA methylation changes were observed among TSS regions, which could mean that gene promoters within heterochromatin regions are highly affected by radiation^56^. In summary, it seems that epigenetic architecture plays a major role in cell response to irradiation. Specifically, it is architecture of heterochromatin associated domains with lower chromatin accessibility which could also explain the propagation of radiation laterally in nuclear layers, since DNA domains are more packed and in closer relationship to each other.

Additionally, there were a few articles highlighting the role of histone marks in the context of GCR or microgravity. Gambacurta et al. discovered that H3K4me3, H3K27me2/3, H3K79me2/3 and H3K9me2/3 are engaged in cellular reprogramming that drives gene expression in blood-derived stem cells during osteoblastic differentiation induced by rapamycin under microgravity^57^. Furthermore, Singh et al. showed that in human T-lymphocyte cells, simulated-microgravity results in DNA hypomethylation^58^. However, gene expression analysis showed a decreased expression of histone deacetylases (HDAC) 1 which should have resulted in increased acetylation of histone H3 which in fact was decreased suggesting regulation of other HDAC. Finally, Koaykul et al. investigated neurogenic differentiation potential of passaged human bone marrow-derived mesenchymal stem cells under conventional and simulated microgravity^59^. Researchers discovered that conventional gravity conditions resulted in higher enrichment of H3K27me3 at neuronal promoters (NF-H and MAP2) when compared to simulated conditions. Contrary, there were no enrichment differences among H3K4me3.

In summary, our study provides novel insights towards epigenetic nuclear architecture. We showed that GCR particles induce unique acute and chronic DNA methylation and gene expression patterns. Moreover, for the first time, we highlighted the role of spatial distribution of key epigenetic modification. Specifically, we point out the major role of heterochromatin associated domains in DNA accessibility and radiation response. Finally, we showed which regions of human genome are the most vulnerable to GCR and thus associated with higher DNA methylation changes. To overcome the main limitation of the study resulting from analyzing similar, but not the same cell types (bronchial epithelial cells and bronchial fibroblasts), we run additional analysis using in vivo models and human tissues. Importantly, all key in vitro findings were successfully validated across more advanced models.

Further research should focus on extensive analysis using various methods discussed in this manuscript. All this should be performed in the same cell type or organism to minimize potential bias which could occur in our analysis. Additionally, it should further investigate the role of various histone modifications which could potentially limit the negative effects of GCR and other radiation types. If successfully treated, it could contribute to development of epigenetically modified preventing strategies against radiation-induced cell damage.

### Supplementary Information

Supplementary Figures.

## Data Availability

This study was done based on publicly available data repositories (Gene Expression Omnibus—https://www.ncbi.nlm.nih.gov/geo/; ENCODE—https://www.encodeproject.org; 4DNucleome—https://www.4dnucleome.org; GeneLab—https://genelab.nasa.gov). Detailed description of each dataset used can be found in methods section of the manuscript. The R code used in the analysis was published on GitHub repository (https://github.com/jakubmie/GCR).
