# Supplementary material for: Chromosomal positioning and epigenetic architecture influence DNA methylation patterns triggered by galactic cosmic radiation
Source: Sci Rep. 2024 Jan 15;14:1324. doi: 10.1038/s41598-024-51756-7 (PMC10789781; doi:10.1038/s41598-024-51756-7)
Supplement: Supplementary file 1 — Supplementary Figures. [file 41598_2024_51756_MOESM1_ESM.pdf]

Supplementatry Figure 1

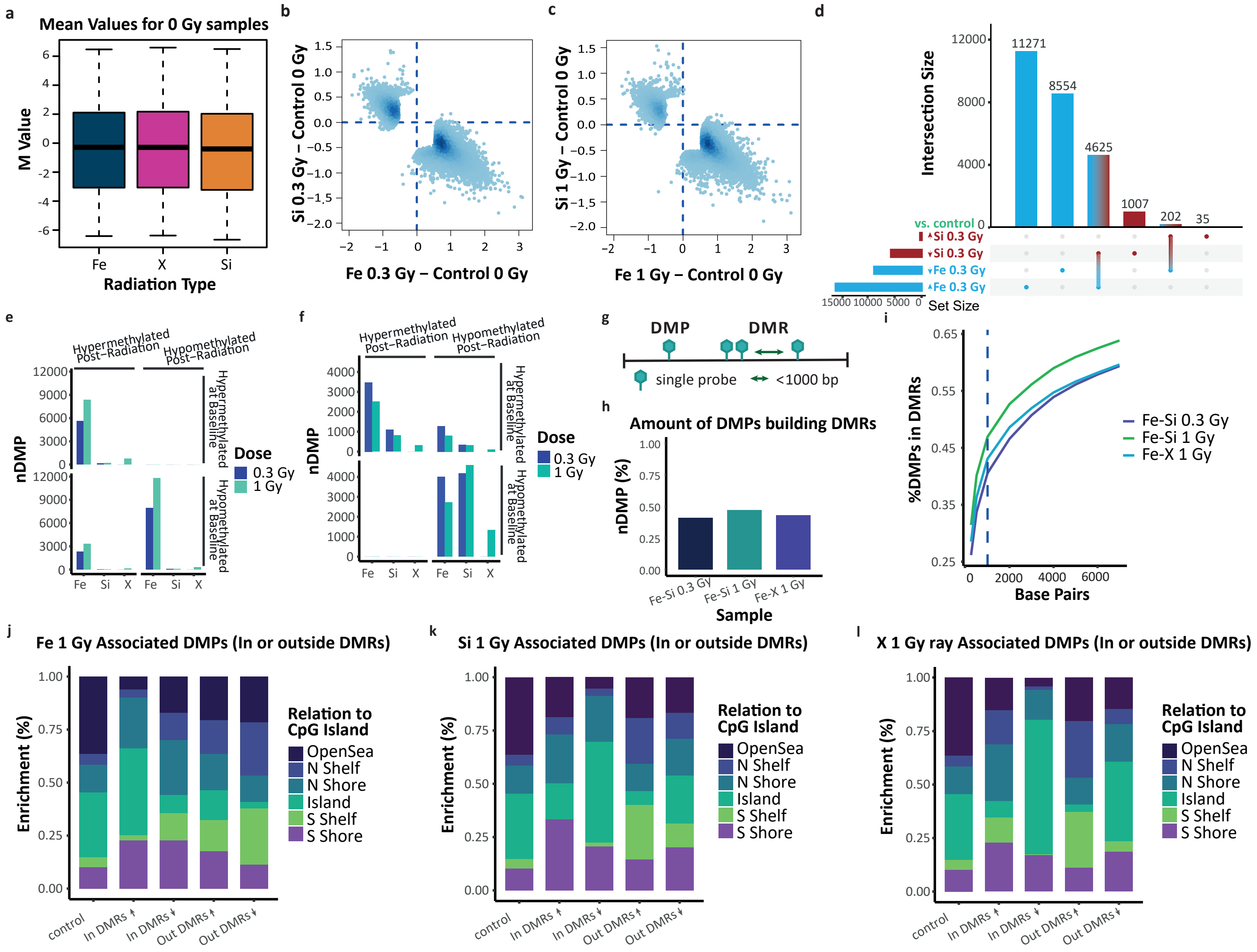

Supplement Figure 1. DNA methylation patterns and differential methylation analysis for 48-hour post-exposure samples. Differentially methylated regions (DMRs) building.

(A) Mean methylation level for non-irradiated samples. **(B-C)** DNA methylation change versus control among DMPs with applied filters (q-value (q < 0.05) and the absolute avDiff between compared samples (avDIiff >= 0.58)). **(D)** The overlay of DMPs which methylation level changed (>= 0.58) versus control between particles. X axis – grouping of individual and overlayed DMPs; Y axis – number of DMPs in each group. **(E-F)** Detailed DNA methylation patterns with inclusion of pre- and post- radiation methylation status. **(G)** The schematic representation of DMRs building. **(H)** Amount of DMPs in DMRs (lambda = 1000) **(I)** Amount of DMRs with different lambda (distance between two DMPs) values applied. **(J-L)** Enrichment of DMPs in CpG island's locations for DMPs inside and outside of DMRs. Shores are up to 2kb from the CpG island; Shelves are from 2kb to 4kb from the CpG island; Opensea refers to isolated regions that do not have a specific designation.

Supplementary figure 2

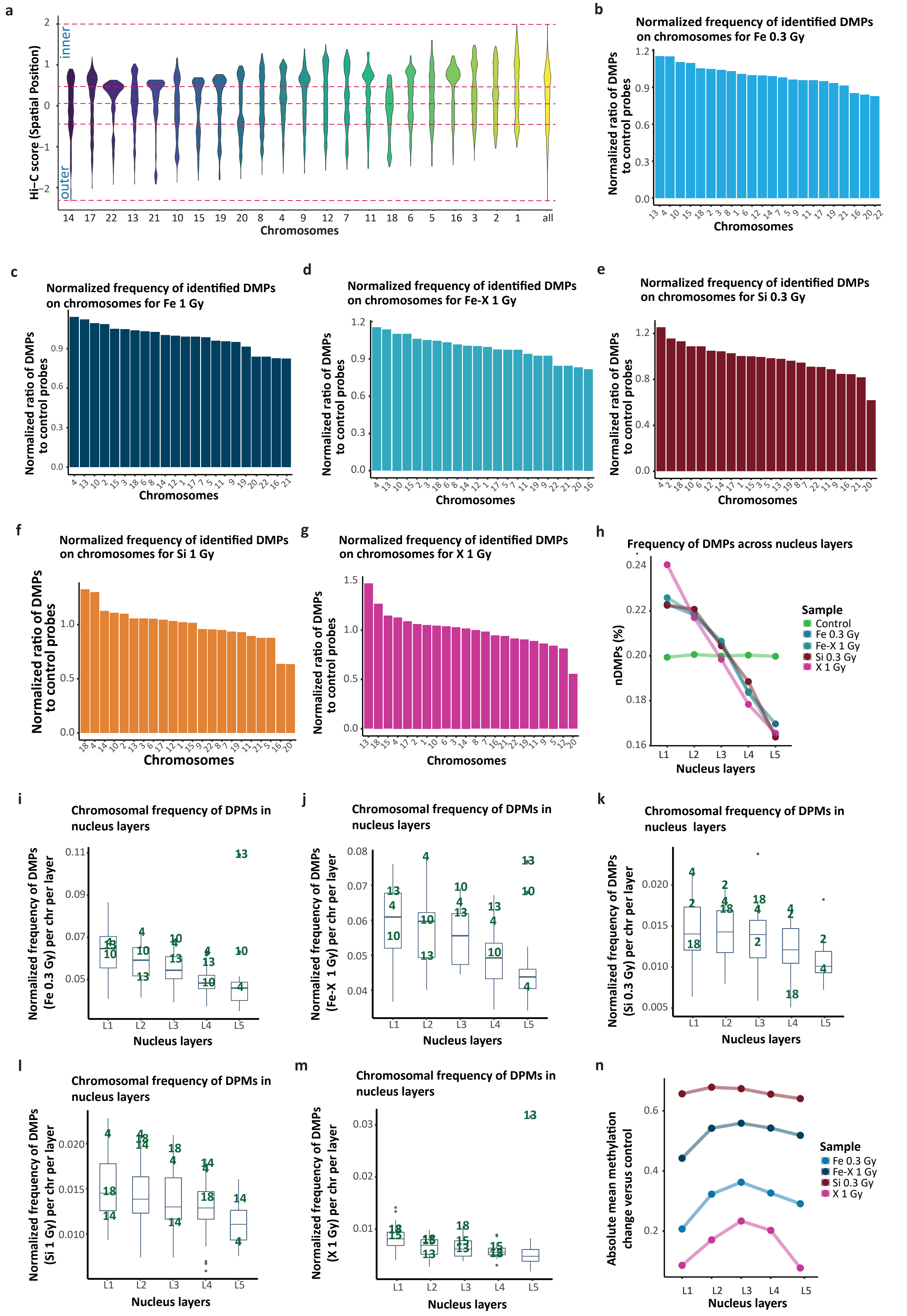

Supplement Figure 2. Radial nucleus architecture and DNA methylation patterns.

(A) Chromosome positioning of non-irradiated primary probes among five nucleus layers (dashed red lines). Chromosomes are grouped from the most externally (chr14) positioned to the most internally (chr1) positioned chromosome. Y axis represents a Hi-C score for individual DMP. **(B-G)** The frequency of DMPs on chromosomes for each exposure. **(H)** The frequency of DMPs and non-irradiated probes among nucleus layers. **(I-M)** Chromosomal frequencies of DMPs in each nucleus layer. Top three chromosomes with the highest overall DMPs frequency are bolded. Boxplots represent data for whole genome. **(N)** Absolute mean DNA methylation change in DMPs versus control among nucleus layers.

Supplementary figure 3

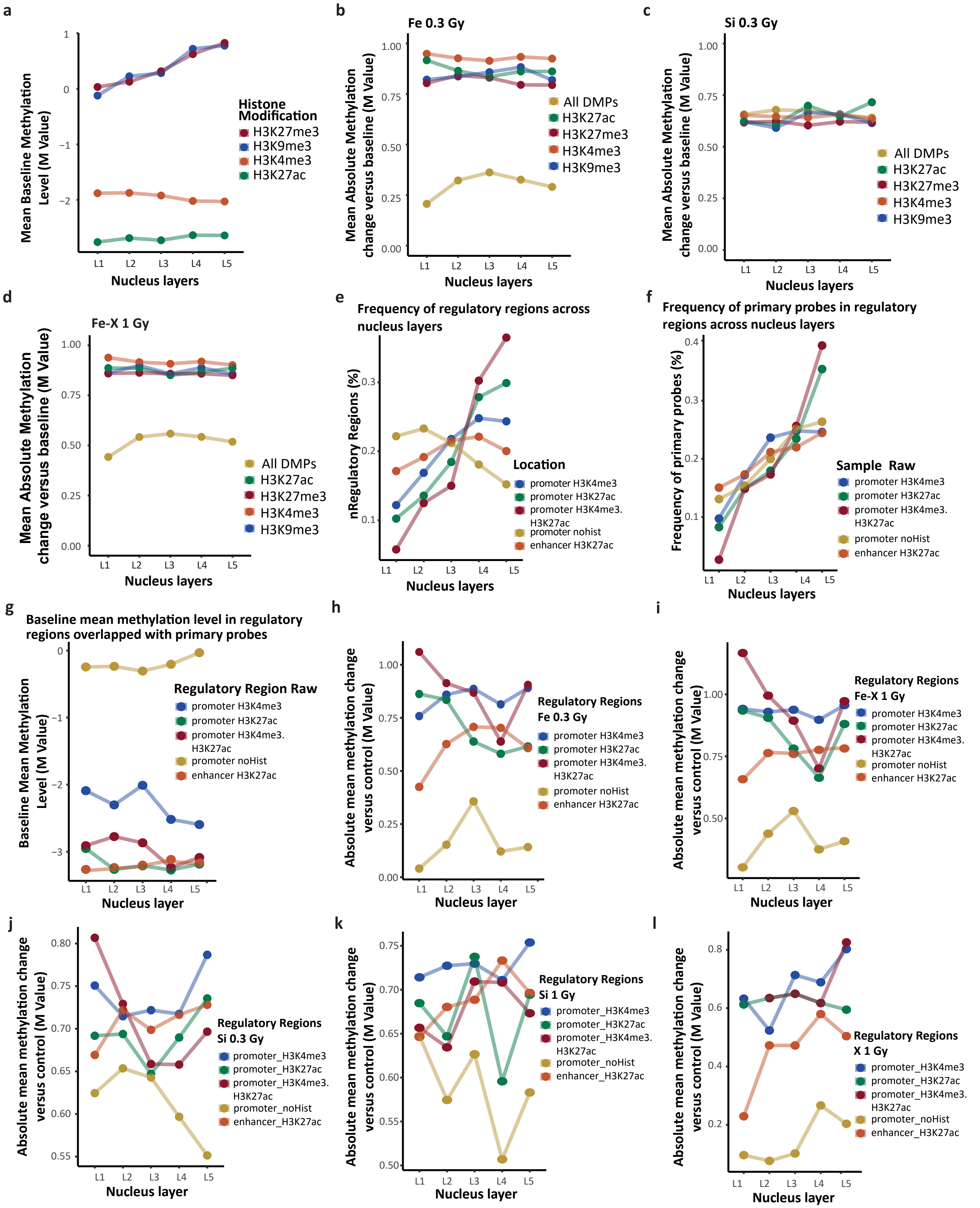

Supplement Figure 3. Radial nucleus architecture of histone modifications.

(A) Mean control methylation level among histone modification peaks overlapped with non-irradiated primary probes. **(B-D)** An absolute mean DNA methylation change versus control among histone modification peaks overlapped with DMPs within nucleus layers. **(E)** The frequency of DMPs in regulatory regions within nucleus layers. **(F)** The frequency of non-irradiated primary probes in regulatory regions within nucleus layers. **(G)** Mean control methylation level among histone associated regulatory regions overlapped with non-irradiated primary probes. **(H-L)** An absolute mean DNA methylation change versus control among histone modification associated regulatory regions overlapped with DMPs within nucleus layers.

Supplementary figure 4

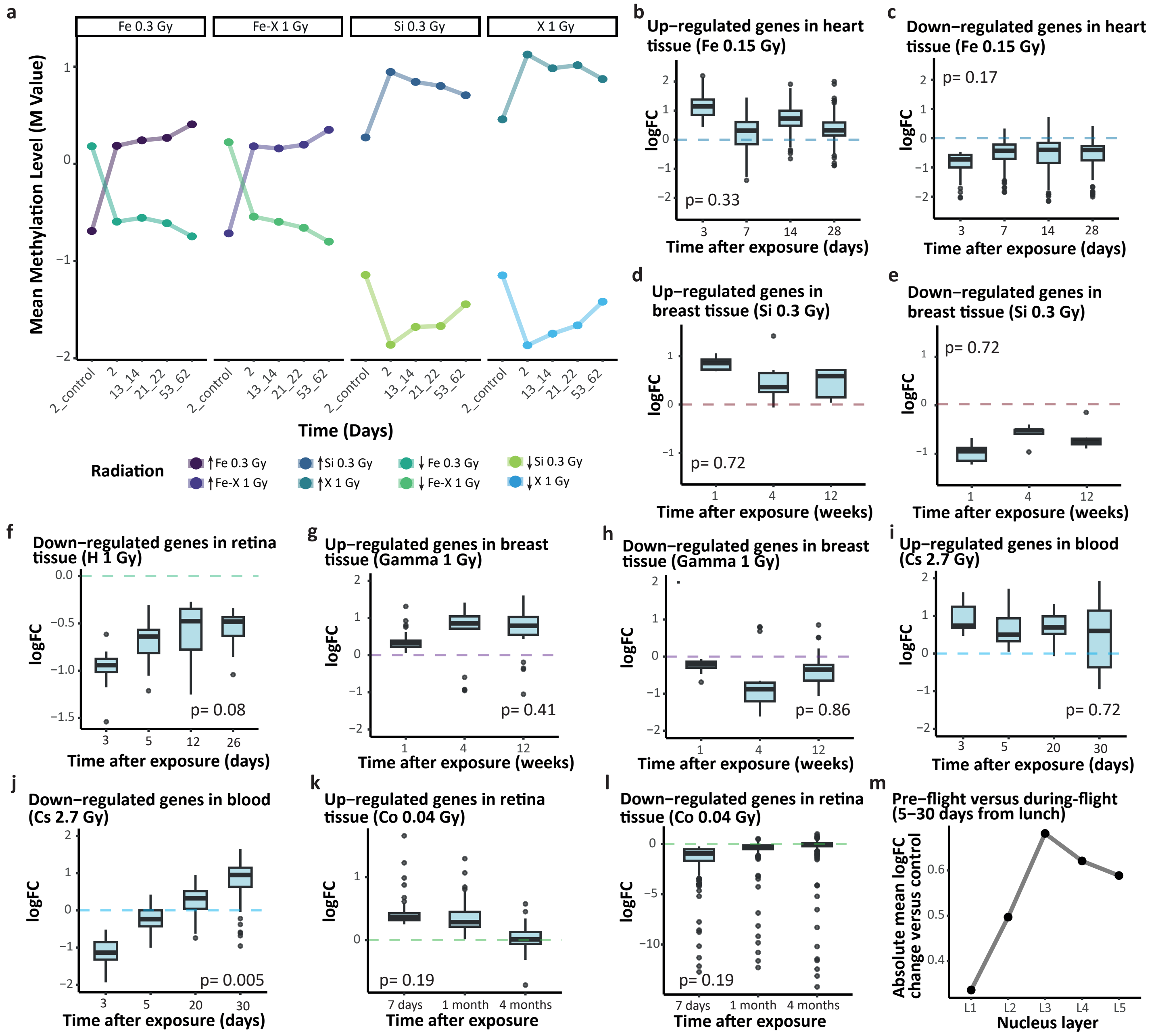

Supplement Figure 4. Persistence of DNA methylation and gene expression change over time. **(A)** Mean DNA methylation change versus control over time with inclusion of direction of methylation change. **(B-L)** The average change of gene expression over time among up-regulated and down-regulated genes. Data presented for distinct tissues and exposures. **(M)** The absolute mean gene expression changes among nucleus layers for all identified genes.
